# Synthesis Mechanism and Thermal Optimization of an Economical Mesoporous Material Using Silica: Implications for the Effective Removal or Delivery of Ibuprofen

**DOI:** 10.1371/journal.pone.0130253

**Published:** 2015-07-10

**Authors:** Shanmuga Kittappa, Mingcan Cui, Malarvili Ramalingam, Shaliza Ibrahim, Jeehyeong Khim, Yeomin Yoon, Shane A. Snyder, Min Jang

**Affiliations:** 1 Department of Civil Engineering, Faculty of Engineering, University of Malaya, Kuala Lumpur, Malaysia; 2 School of Civil, Environmental, and Architectural Engineering, Korea University, Seoul, Republic of Korea; 3 Department of Chemistry, Jalan Sultan Petaling Jaya, Selangor, Malaysia; 4 Department of Civil and Environmental Engineering, University of South Carolina, Columbia, South Carolina, United States of America; 5 Department of Chemical and Environmental Engineering, University of Arizona, Tucson, Arizona, United States of America; 6 Nanotechnology and Catalysis Research Centre (NANOCAT), University of Malaya, Kuala Lumpur, Malaysia; University of Quebec at Trois-Rivieres, CANADA

## Abstract

Mesoporous silica materials (MSMs) were synthesized economically using silica (SiO_2_) as a precursor via a modified alkaline fusion method. The MSM prepared at 500°C (MSM–500) had the highest surface area, pore size, and volume, and the results of isotherms and the kinetics of ibuprofen (IBP) removal indicated that MSM–500 had the highest sorption capacity and fastest removal speed *vs*. SBA–15 and zeolite. Compared with commercial granular activated carbon (GAC), MSM–500 had a ~100 times higher sorption rate at neutral pH. IBP uptake by MSM–500 was thermodynamically favorable at room temperature, which was interpreted as indicating relatively weak bonding because the entropy (∆_*adsS*_, –0.07 J mol^–1^ K^–1^) was much smaller. Five times recycling tests revealed that MSM–500 had 83–87% recovery efficiencies and slower uptake speeds due to slight deformation of the outer pore structure. In the IBP delivery test, MSM–500 drug loading was 41%, higher than the reported value of SBA–15 (31%). The *in vitro* release of IBP was faster, almost 100%, reaching equilibrium within a few hours, indicating its effective loading and unloading characteristics. A cost analysis study revealed that the MSM was ~10–70 times cheaper than any other mesoporous silica material for the removal or delivery of IBP.

## Introduction

Mesoporous silica materials (MSMs) have large, ordered pores, ranging from 2 to 50 nm, as classified by the International Union of Pure and Applied Chemistry (IUPAC) [1, 2]. MSMs have thick framework walls with interconnected channel structures and a high surface area that can provide superior properties for adsorption, catalysis, and sensing [1, 3, 4]. Because of evenly distributed pores (6–10 nm in size), MSMs readily allow organic molecules, including pharmaceuticals, to penetrate through their pores, resulting in less pore diffusion [1, 5].

In addition to environmental applications (e.g., water treatment), mesoporous materials have emerged as suitable candidates for drug delivery systems and other biomedical applications [6, 7]. Reference materials such as SBA–15, MCM–41, and MCM–48 have been studied extensively for the loading and unloading of pharmaceuticals [3, 4, 7–9]. However, these media face a major hurdle in actual application due to the use of expensive organometallic chemicals, such as tetraethyl orthosilicate (TEOS, Si(OC_2_H_5_)_4_) [3, 10], which is used as a cross-linking agent in the synthesis process [2, 11]. Thus, a more economical approach is essential for synthesizing MSMs if they are to be used in pharmaceutical removal or delivery processes.

Ibuprofen (IBP) was selected as a model drug for this study because it is one of the most widely consumed drugs worldwide; it is classified as a nonsteroidal anti-inflammatory drug (NSAID) [12, 13]. Indeed, due to the high concentration of IBP in water sources, it has been classified as a pharmaceutical pollutant by the World Health Organization (WHO). Moreover, IBP has been studied widely for loading and unloading purposes as a model drug [14].

To synthesize MSMs for IBP removal or delivery economically, we developed a modified alkaline fusion method, which broadens the choice of framework precursors. For the first time, we synthesized MSMs using SiO_2_ as a framework precursor, replacing TEOS. The main objectives of this investigation were to (i) assess the effects of thermal treatment (calcination) through characterization using X-ray diffraction (XRD), transmission electron microscopy (TEM), Fourier transform infrared spectroscopy (FTIR), and N_2_ gas isotherm; (ii) assess the sorption capacities and rates of IBP in multi-recycle runs; (iii) study IBP loading and unloading by MSMs; and finally (iii) determine the adsorption mechanism.

## Materials and Methods

Pluronic P123 (EO_20_PO_70_EO_20_) and SiO_2_ were obtained from Sigma-Aldrich (St. Louis, MO, USA) and R&M Chemicals (Edmonton, AB, Canada), respectively. Sodium hydroxide (NaOH) and hydrochloric acid (HCl) (37%) were purchased from Merck (Darmstadt, Germany). Analytical grade IBP was supplied by Alfa Aesar (Royston, Hertfordshire, UK).

### Synthesis method of the MSMs

MSMs were synthesized by reacting Pluronic P123 with SiO_2_ in the presence of NaOH and HCl. SiO_2_ (1 M) was dissolved in a 1 M NaOH solution and stirred using a magnetic stirrer at 45°C for 20 h. The pore-templating agent was prepared separately using 4 g of Pluronic P123, dissolved in 120 mL of a 2 M HCl solution with continuous stirring at 45°C. The two solutions were then mixed and stirred for 3 h at 45°C, and then for an additional 12 h at room temperature. The molar ratio of chemicals used in the synthesis of the MSM was 1 SiO_2_:1 NaOH: 5.28 HCl: 0.015 Pluronic: 200 H_2_O. The solution was then aged in a Teflon bottle at 90°C for 20 h. The precipitated solid product was recovered by filtering it using a 0.45 μm pore size cellulose acetate membrane filter. Finally, the material was washed with deionized (DI) water and ethanol (50%) and dried at 60°C for 24 h. A Western furnace was used to calcine the dried samples at temperatures ranging from 500 to 900°C for 4–6 h.

### Characterization of the MSMs

XRD analysis was performed using an X-ray diffractometer (Empyrean; PANalytical, Almelo, The Netherlands). The samples were scanned at 2*θ* from 0.5 to 2.5° using a step size of 0.0070° and a scanning time of 19.9260 s. Nitrogen adsorption and desorption isotherms were measured using a TriStar II 3020 system (Micromeritics, Norcross, GA, USA). The samples were analyzed at 77.35 K. The adsorption and desorption data were calculated using Brunauer–Emmett–Teller (BET) theory to determine the specific surface area of the samples, and the pore-size distributions and pore volumes were determined using the Barrett–Joyner–Halenda (BJH) theory. Infrared (IR) spectra were obtained using a NICOLET IS 10 spectrometer (Thermo Fisher Scientific, Waltham, MA, USA). Microscopic images of nanoscale pore structures were taken using a transmission electron microscope (HT 7700 TEM; Hitachi, Tokyo, Japan) at 120 kV.

### Preparation of IBP stock solution

The required amount of IBP powder (200 mg) was dissolved in 10% methanol (CH_3_OH; Thermo Fisher Scientific) and dissolved in DI water in a 250 mL volumetric flask. It was then stirred for 12 h, sonicated for 2 h, and filtered using a 0.45 μm pore-size cellulose acetate membrane filter. A sodium chloride (NaCl) solution was added to the filtrate to create ionic strength (0.01 M) in the solution. The pH was adjusted to 7 using a sodium phosphate (Na_3_PO_4_) solution.

### Isotherms and kinetics of IBP removal by the MSMs

Adsorption isotherms and kinetics were determined for four synthesized MSMs calcined at 500, 600, 700, and 900°C, which were denoted as MSM–500,–600,–700 and–900, respectively.

#### Isotherms

In 20 mL vials, 10–500 mg of MSM and 10 mL of IBP solution (150 mg L^–1^) were added together. The vials were placed in an electric shaker and agitated at 250 rpm and 25°C. The suspension was filtered using a 0.45 μm pore-size cellulose acetate membrane filter, and the filtrate was analyzed for IBP.

The data were fitted with the Langmuir and Freundlich isotherms. When the adsorption is retained on a uniform monolayer surface, the Langmuir model fits the data of the isotherm. The maximum adsorption capacity is achieved when all sorption sites are saturated. The linear form of the Langmuir model can be written as

$$q_{eq}=\frac{Q_{\text{max}}K_{L}C_{eq}}{1+K_{L}C_{eq}}$$ (1)

where *q*_eq_ is the amount of solute adsorbed per unit weight of adsorbent (mg g^–1^) at equilibrium, *C*_eq_ is the equilibrium concentration of the solute in the bulk solution (mg L^–1^), *Q*_max_ is the maximum adsorption capacity (mg g^–1^), and *K*_L_ is the Langmuir constant related to the energy of adsorption.

Assuming that the adsorption is retained on the heterogeneous surface of the adsorbent, the Freundlich model fits the isotherm data better. In the Freundlich model, chemisorption and physisorption are pertinent to monolayer and multilayer adsorption, respectively. The linear form of the Freundlich equation can be expressed as

$$\text{log}\;q_{eq}=\text{log}\;K_{F}+\frac{1}{n}\text{log}\;C_{eq}$$ (2)

where *K*_F_ and *n* are Freundlich isotherm constants related to the adsorption capacity and adsorption intensity, respectively.

#### Kinetics

IBP solutions (200 mL) with concentrations of 100 mg L^–1^ were added to four separate beakers in which 1,000 mg of MSM had been pre-added. These four beakers were stirred constantly at 200 rpm for 200 min at room temperature. At timed intervals, samples from each of the four beakers were collected, filtered, and analyzed for IBP. After obtaining the kinetic data, a pseudo-second-order kinetic model equation was used to obtain the kinetic constants of the reaction,

$$\frac{t}{q_{t}}=\frac{1}{K_{2}q_{eq}{}^{2}}+\frac{t}{q_{eq}}$$ (3)

where *K*_2_ is the rate constant of pseudo-second-order adsorption (g mg^–1^ min^–1^), *K*_2_ *q*_eq_^2^ or *v*_0_ (mg g^–1^ min^–1^) is the initial adsorption rate, and *q*_t_ is the amount of adsorbate adsorbed at time *t* (min).

#### Thermodynamics

The kinetics of IBP uptake by MSM-500 were examined at 299, 309, and 319 K. Thermodynamic values, such as the Gibbs free energy (*Δ*_*ads*_ *G* ^*0*^), enthalpy (*Δ*_*ads*_ *H* ^*0*^), and entropy (*Δ*_*ads*_ *S* ^*0*^), were determined using the kinetic data. This adsorption process is represented by a reversible heterogeneous equilibrium: IBP in solution ↔ IBP adsorbed.

The thermodynamic calculation was adapted from a mechanism and calculation reported previously [15]. The equilibrium constant is defined using the following equation [16, 17]

$$K^{0}=\frac{q_{e}}{C_{s}}$$ (4)

where *q*_*e*_ and *C*_*s*_ are concentrations of adsorbed IBP and in solution at equilibrium, respectively. The *K* value was substituted to acquire *Δ*_*ads*_ *G* ^*0*^, *Δ*_*ads*_ *H* ^*0*^, and *Δ*_*ads*_ *S* ^*0*^ using the following equations,

$$\Delta_{ads}G^{o}=-RT\text{ln}K^{0}$$ (5)

$$\text{ln}\;K^{0}=\frac{\Delta_{ads}S^{0}}{R}-\frac{\Delta_{ads}H^{0}}{RT}$$ (6)

where R and T are the gas constant and absolute temperature, respectively. *∆*_*ads*_ *H* ^*0*^ and *∆*_*ads*_ *S* ^*0*^ were obtained from the slope and intercept of the line of ln *K vs*. 1/T.

#### Regeneration

MSM–500 was used to test the regeneration of MSM for IBP adsorption. After being used for the first adsorption test, MSM–500 was filtered, washed in methanol for 4–6 h, and dried overnight in an oven. The same medium was reused for four subsequent adsorption tests.

### Drug loading and in vitro drug release

To load IBP, 0.2 g of MSM–500 was added to a hexane solution containing 30 mg mL^–1^ IBP. The powder-containing solution was soaked for 2 days and stirred at 150 rpm at room temperature until the concentration in the solution remained constant. The loaded amount of IBP was determined using the difference in the initial and final IBP concentration. The powder was then washed with the hexane solution and dried under vacuum.

The *in vitro* drug release test was conducted by soaking 0.2 g of IBP containing MSM–500 in 100 mL of phosphate-buffered saline (PBS) at pH 7.4. The solution was stirred at 150 rpm and kept at 36.9°C, similar to body temperature. At each time point, 3 mL of solution was extracted and analyzed, and an equal volume of fresh solution was replaced. The IBP was analyzed to assess the release of IBP. The test was also repeated with the PBS solution at a lower pH, i.e., 4–6.

### Analysis of IBP

IBP was analyzed using a UV 2600 spectrophotometer (Shimadzu, Otsu, Japan). A standard IBP solution was subjected to a full-spectrum scan, ranging from 900 to 0 nm wavelengths, and maximum absorbance (*λ*_max_) was detected at 264 nm. A calibration curve, which was obtained by analyzing various concentrations of the prepared IBP standard solution, showed good linearity, with a determination coefficient (*R* ^2^) of 0.9997.

## Results and Discussion

### Thermal effect analysis and characterization of the MSMs

**Fig 1:**
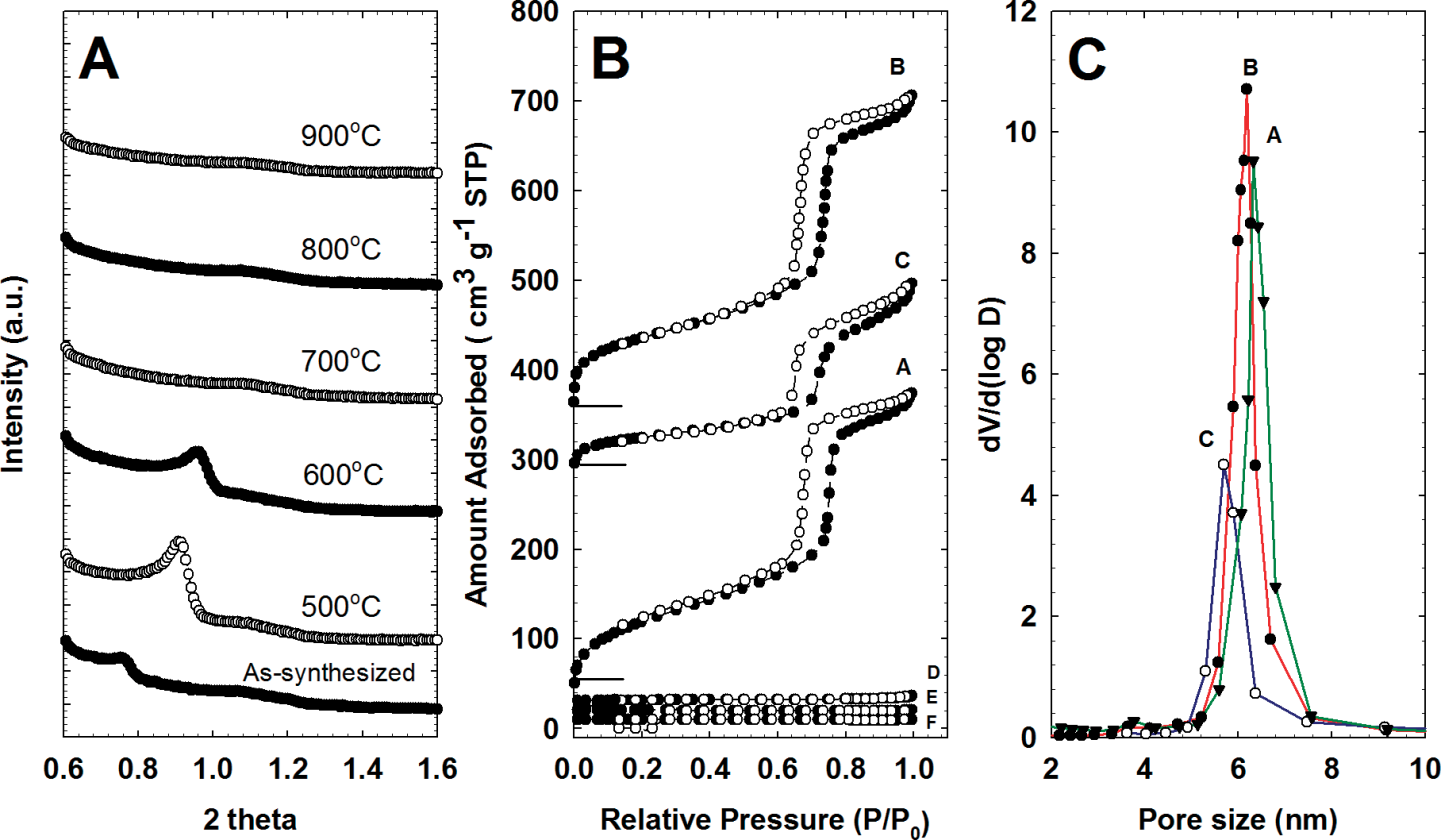
(A) XRD patterns of as-synthesized mesoporous material and MSMs calcined at different temperatures, (B) the N_2_ sorption isotherm, and (C) pore-size distribution of the MSMs (A: MSM-as synthesized, B: MSM-500, C: MSM-600, D: MSM-700, E: MSM-800, and F: MSM-900).

Fig 1(A) shows a low-angle XRD diffraction pattern of selected MSMs. Among the synthesized media, as-synthesized MSM, MSM–500, and MSM–600 showed well-resolved peaks indexed at the *d*(100) reflection [1, 7, 18]. Additionally, MSM–500 had two more weak peaks at 2*θ* angles of 1.08 and 1.58°, indexed at *d*(110) and *d*(200), respectively. These types of XRD reflection patterns can be assigned to a well-ordered mesoporous structure with *P*6*mm* hexagonal symmetry [1, 2, 19].

The XRD results indicated that MSM–500 had the highest peak intensity compared with the other MSMs, reflecting the formation of more uniform mesopores [4, 19]. The shifting of peaks to lower 2*θ* values with higher calcination temperature shows an increase in the pore sizes of the MSMs, indicating the possibility that MSM–500 had larger pores than MSM–600 and –700. However, MSMs calcined at >700°C showed *no* XRD diffraction peak, demonstrating the collapse of the pore structure [19]. This phenomenon was investigated further using N_2_ adsorption–desorption analysis.

Park et al. reported that synthesis of mesoporous materials using silica glass at pH >10 yielded a white gel that transformed into a mesoporous material due to a hydrothermal process [20]. However, in the current method, we synthesized the MSMs under acidic conditions (pH <1), whereby a clear white precipitate was formed immediately after the silicate (HSiO_3_^–^) and acidified Pluronic P123 solutions were mixed together. The precipitate was treated using a hydrothermal process, in which it was heated and condensed to form the MSM. This method allows the excess hydrogen (H^+^) and chlorine (Cl^–^) to coordinate electrostatically between the nonionic pore-templating agent (S^o^) and the hydrogen-associated silicic acid (I^o^, Si(OH)_4_), which may finally lead to the formation of S^o^H^+^Cl^–^I^o^. Given these observations, the reaction mechanism of this synthesis can be represented using reactions 1–3.

First, the mixing of NaOH and SiO_2_ may lead to the formation of silicate ions (HSiO_3_^–^) (R1).

$${\text{SiO}}_{2}+\text{NaOH}\to {\text{Na}}^{+}+{\text{HSiO}}_{3}^{-}$$ (R1)

Second, when both the silicate and acidic Pluronic solution are mixed together, silicic acid (Si(OH)_4_) is formed under acidic conditions (R2).

$${\text{HSiO}}_{3}^{-}+\text{HCl}+{\text{H}}_{2}\text{O}\to {\text{Si}(\text{OH})}_{4}+{\text{Cl}}^{-}$$ (R2)

Finally, through a hydrothermal process, two silicic acids are condensed and form a silicate dimer, which further polymerizes to form the framework of the MSM (R3).

$${\text{Si}(\text{OH})}_{4}+{\text{Si}(\text{OH})}_{4}\underset{\;\Delta 90{}^{\circ}\text{C}\;}{\to }{\text{Si}}_{2}\text{O}{\left(\text{OH}\right)}_{6}+{\text{H}}_{2}\text{O}$$ (R3)

Fig 1(B) shows the N_2_ adsorption–desorption isotherms of the various MSMs. According to the IUPAC classification, the as-synthesized MSM, MSM–500, and MSM–600 had a type-IV sorption pattern [21, 22], whereas MSMs calcined at >700°C showed no sorption profile. At a relative pressure of *P/P*_o_ < 0.2, MSM–500 had more primary micropores (<2 nm) than MSM–600. Reversible sorption of N_2_ gas for MSM–500 and–600 occurred up to a *P/P*_o_ of 0.6, whereas that for as-synthesized MSM happened much earlier, at a *P/P*_o_ of 0.2. These results indicate that MSM–500 and–600 had more uniform pores with a hysteresis loop pattern type H_1_, similar to SBA–15 [23, 24]. However, this MSM behavior differed from that of MCM–41, in which the adsorption and desorption isotherm process were fully reversible [25].

Using the BJH method, the pore-size distribution was obtained for as-synthesized MSM, MSM–500, and–600 (Fig 1(C)). These results showed that MSM–500 had the narrowest pore-size distribution, with a primary peak at 6.2 nm, while MSM–600 displayed a broader distribution and a primary peak at 5.6 nm. This indicates that MSM–500 had a larger pore structure and pore volume than MSM––600, consistent with the XRD results in Fig 1(A).

The pore structure characteristics of MSMs and other referenced media are summarized in Table 1. Among the calcined MSMs, MSM–500 had the highest BET specific surface area (310 m^2^ g^–1^) and pore volume (0.541 cm^3^ g^–1^). The BET specific surface area and pore volume decreased substantially when the calcination temperature increased from 500 to 900°C. A significant decrease was noted at 700°C, with the BET specific surface area reduced to <10 m^2^ g^–1^. Generally, the MSMs synthesized had smaller BET specific surface areas and pore volumes than reported for SBA–15 [9, 18].

**Table 1:** Structural parameters of as-synthesized and calcined MSMs.

	*d-*	Mean	Wall	Surface	*V*_t_ ^e^	*V*_m_
	spacing^a^	pore	thickness^c^	area^d^	Pore volume	Micropore
	(nm)	diameter^b^ (nm)	(nm)	(m^2^ g^–1^)	(cm^3^ g^–1^)	(cm^3^ g^–1^)
MSM as-synthesized	11.9	5.78	7.9	326	0.541	0.025
MSM-500	9.68	6.26	4.9	310	0.565	0.011
MSM-600	9.33	6.93	3.8	160	0.339	0.011
MSM-700	n/a	4.76	n/a	6.3	0.0089	0.0069
MSM-800	n/a	1.98	n/a	1.7	0.0039	0.0029
MSM-900	n/a	n/a	n/a	0.7	0.0006	0.0005

^a^ (100) interplanar spacing.

^b^Calculated from desorption of the N_2_ isotherm.

^c^Determined from the difference between the unit cell parameter (*a*_o_ = 2d_100_/√3) and the frame work pore size.

^d^BET specific surface area calculated from adsorption of the N_2_ isotherm.

^*e*^ *V*_t_ = total pore volume calculated using BJH.

The corresponding unit cell parameter (*a*_o_) and mean BJH desorption pore diameter were used to determine the wall thickness of the MSMs. The wall thickness was calculated as the difference between *a*_o_, where *a*_o_ = 2 × *d*(100)/$\sqrt{3}$, and the mean BJH desorption pore diameter [2, 26]. The mean BJH desorption pore diameter increased during the calcination process at 500°C and 600°C (6–7 nm) for MSMs and was smaller than that of SBA–15 (~10 nm). At the same temperature, the MSMs *d*-spacing and wall thickness decreased from 11.9 to 9.3 nm and 7.6 to 3.3 nm, respectively. This reduction in wall thickness is a result of silicate condensation during calcination [27]. Calcination at temperatures >700°C caused a significant decrease in the wall thickness and the framework to collapse [2, 28].

In this study, MSMs were found to have lower thermal stability (up to 600°C), which could be due to the formation of weaker bonds between the condensed silica walls. In contrast, Tung et al. reported that SBA––15 and a few other mesoporous silica-based materials had high thermal stabilities of up to 850°C [1].

### Adsorption isotherms of IBP by the MSMs

**Fig 2:**
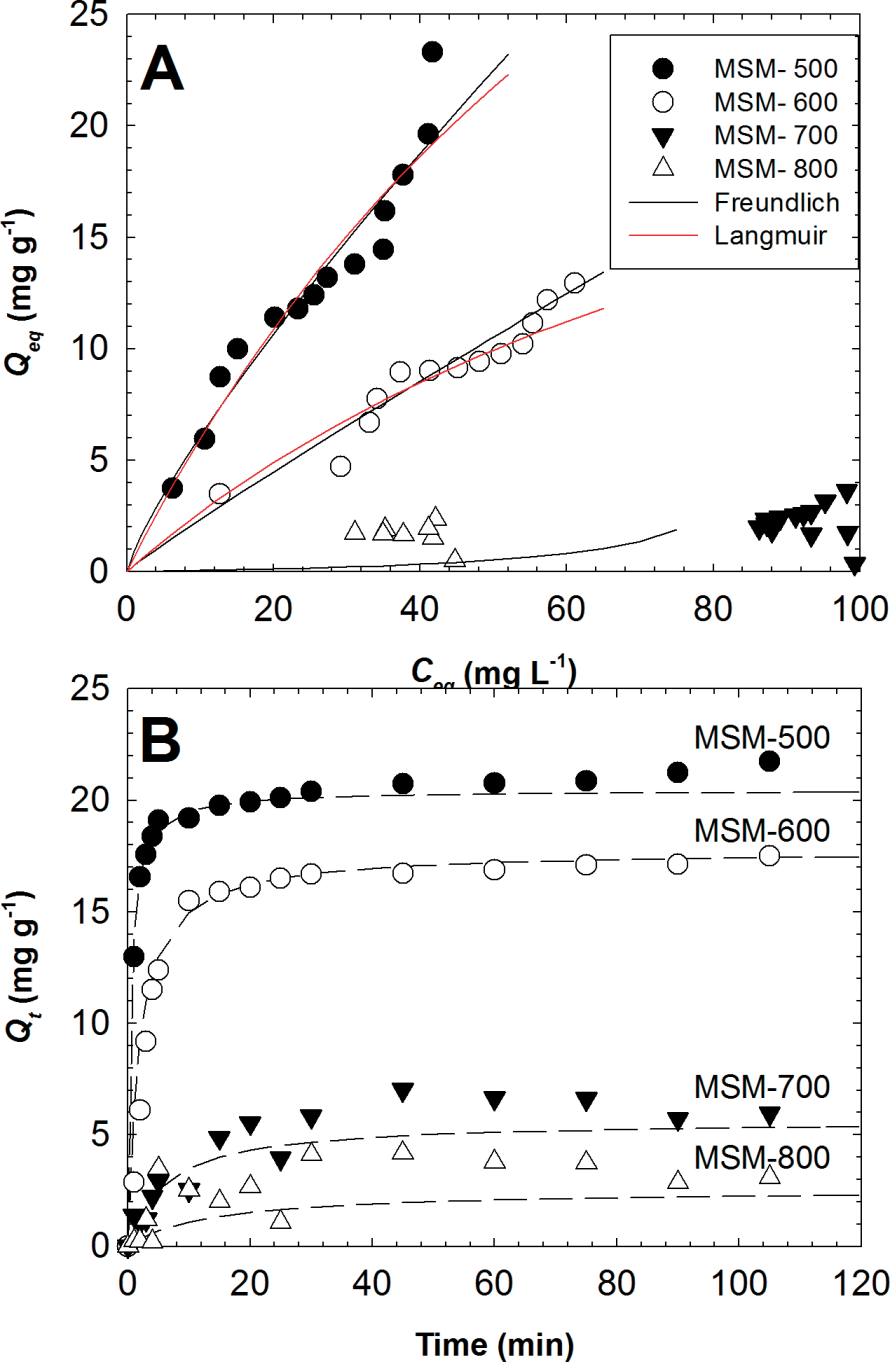
(A) Adsorption isotherms of the MSMs calcined at different temperatures. (B) Kinetics of IBP uptake by MSMs calcined at different temperatures.

The IBP adsorption isotherms were determined using MSMs calcined at different temperatures (Fig 2(A)). The *Q*_eq_ of IBP by MSM–500 and–600 increased with increasing *C*_eq_, although differences were observed in the adsorption capacity. However, MSM–700 and–900 showed no significant adsorption, consistent with the physical property analyses using XRD and N_2_ adsorption–desorption analysis. To better understand the adsorption phenomenon of these MSMs, Langmuir and Freundlich isotherms were fitted with the experimental data of MSM–500 and–600.

The Langmuir and Freundlich parameters, along with determination coefficients (*R* ^2^) of the linear plots, were calculated. The results are presented in Table 2. The adsorption capacities (*Q*_max_) for MSM–500 and–600 varied considerably: 64.8 mg g^–1^ for MSM–500 and 31.9 mg g^–1^ for MSM–600.

**Table 2:** Isotherm parameters obtained by fitting equilibrium data with the Freundlich and Langmuir isotherms for the adsorption of IBP on MSMs.

Sample		Langmuir equation			Freundlich Equation			Ref.
	pH	*K*_L_ (L mg^–1^)	*q*_m_ (mg g^–1^)	*R* ^2^	^a^ *k*_F_	1/*n*	*R* ^2^	
MSM-500	7	0.010	64.8	0.97	0.93	0.82	0.95	This study
MSM-600	7	0.009	31.9	0.74	0.27	0.93	0.75	This study
SBA-15	5	13.330	0.41	0.97	1.50	0.78	0.97	[1]
MCM-41	–	0.328	–	–	–	–	–	[7]
GAC	–	n/a	n/a	n/a	0.072	0.87	0.99	[7]
CAC	~4	0.262	153.2	0.999	39.1	0.38	0.890	[29]
CPAC	~4	0.112	416.7	0.998	56.9	0.48	0.919	[29]
AC-olive waste	4.1	0.55	12.6	0.971	6.58	0.34	0.956	[30]
Commercial GAC	–	0.61	160	–	–	4	–	[31]
Cork-Based AC	4	0.356	139.2	–	36.6	0.303	–	[32]

^a^ *k*_F_ in mg^1–1/n^ L^1/n^ g^–1^, *K*_L_: Langmuir constant, *k*_F_ and *n*: Freundlich constants, *q*_max_: maximum amount of adsorbate.

As the value of *K*_L_ increased, the adsorption affinity of IBP by the adsorbent increased. The results show that MSM–500 had a higher affinity than MSM–600, with a higher *K*_L_ value (Table 2).

However, the adsorption tests of MSMs were conducted at neutral pH, so comparing the results with reported *K*_L_ values measured under acidic conditions is difficult.

At higher pH, two important factors, ionization and surface charge of the adsorbent, can affect the adsorption process. The acid dissociation of IBP molecules occurs at a pH higher than the pK_a_ value (4.9), which may increase IBP solubility. As a result, a weaker interaction between the MSM surface and IBP would occur, and the adsorption capacity would decrease significantly. However, the *Q*_max_ of MSM–500 was higher than the value reported for SBA–15.

As a function of adsorption strength, the constant values of 1/*n* obtained from the Freundlich model for IBP removal by MSM–500 and–600 were 0.82 and 0.93, respectively. These values were larger than the constant values of other media except commercial granular activated carbon (GAC; Table 2). If 1*/n* equals 1, then the partition between the two phases is considered independent of the concentration. A value of 1/*n* > 1 demonstrates “normal” adsorption, while a value of 1/*n* < 1 indicates cooperative adsorption. Thus, based on the evaluation of 1/*n*, the adsorptive removal of IBP by MSM–500 and–600 can be concluded to be favorable [33, 34].

The adsorption densities per unit surface area for MSM–500 and–600 were similar, 0.21 and 0.20 mg m^–2^, respectively. These values were comparable to the reported value (0.28–0.32 mg m^–2^) for SBA–15 [7, 8].

### Adsorptive kinetics of IBP by the MSMs

Fig 2(B) shows the kinetics of IBP removal by the MSMs at pH 7. Rapid adsorption of IBP by MSM–500 and–600 was observed for the first 5 min, reaching a plateau after 15 min. After 60 min, the IBP adsorption increased again, suggesting the possibility of IBP molecules forming a dimer [7, 19, 35]. Thus, the contact time needed for MSM–500 and–600 was much shorter than that of activated carbon (AC), which requires ~2 h [29]. The faster adsorption of MSM–500 and–600 may relate to their pore structures. MSMs have mostly mesopores and only 3–5% micropores, while AC has predominantly micropores (as high as 70%), leading to slow diffusion. The pore size of MSMs (~7 nm) is uniform and large, and sufficient for the penetration of IBP molecules (~1 nm). The pore size of MSMs allows the IBP molecules to diffuse readily and adsorb onto the surface of MSMs within a short time, which indicates that MSM–500 and–600 may be advantageous in terms of designing a compact water treatment system, enhancing their economical use commercially. Furthermore, the homogenous pore structures may make them suitable candidates for biomedical applications as drug carrier media.

The adsorption kinetics were investigated using a pseudo-second-order kinetic model, in which *t/q*_*t*_ *vs*. *t* was plotted to obtain the rate parameters. The results showed that the fit of the model to the data had a high determination coefficient (0.99).

Table 3 lists the important parameters obtained from the current kinetic study and other references.

**Table 3:** Kinetic parameters obtained after fitting to the pseudo-second-order model.

Sample	*C*_0_ (mg dm^–3^)	pH	*K*_2_ (g mg^–1^ min^–1^)	*R* ^2^	*K*_*2*_ *q*_*eq*_^*2*^ or *v*_*0*_ (mg g^–1^ min^–1^)	*q*_e,calc._ (mg g^–1^)	Ref.
MSM-500	100	7	0.10	0.999	43.5	20.4	This study
MSM-600	100	7	0.03	0.999	9.58	17.7	This study
MSM-700	100	7	0.02	0.965	0.92	5.6	This study
MSM-900	100	7	0.01	0.622	0.19	2.5	This study
CAC	90	~4	0.07	0.999	834	112.4	[36]
CPAC	90	~4	0.23	0.999	2500	106.4	[36]
Zeolites MNCZ	0.1	7	0.214	0.999	–	0.098	[37]
Commercial GAC	–	7	0.0011	0.952	–	69.95	[31]
Cork-based GAC	90	4	0.07	0.999	834	112.4	[32]

The *K*_2_ values for MSM–500 and–600 were 0.10 and 0.03 g mg^–1^ min^–1^, respectively. The higher *K*_2_ value for MSM–500 indicates that the medium was much faster in adsorbing IBP than the other MSMs. Although several types of AC have higher *K*_2_ values than MSMs (Table 3), most of the tests were conducted under acidic conditions (pH ~4). At pH 7, MSM–500 had a ~100 times higher sorption speed than commercial GAC, which had a *K*_2_ value of 0.0011 g mg^–1^ min^–1^. Zeolites had a higher *K*_2_ value than the MSMs, but the *Q*_max_ was only 0.0976 mg g^–1^.

### Thermodynamics

**Fig 3:**
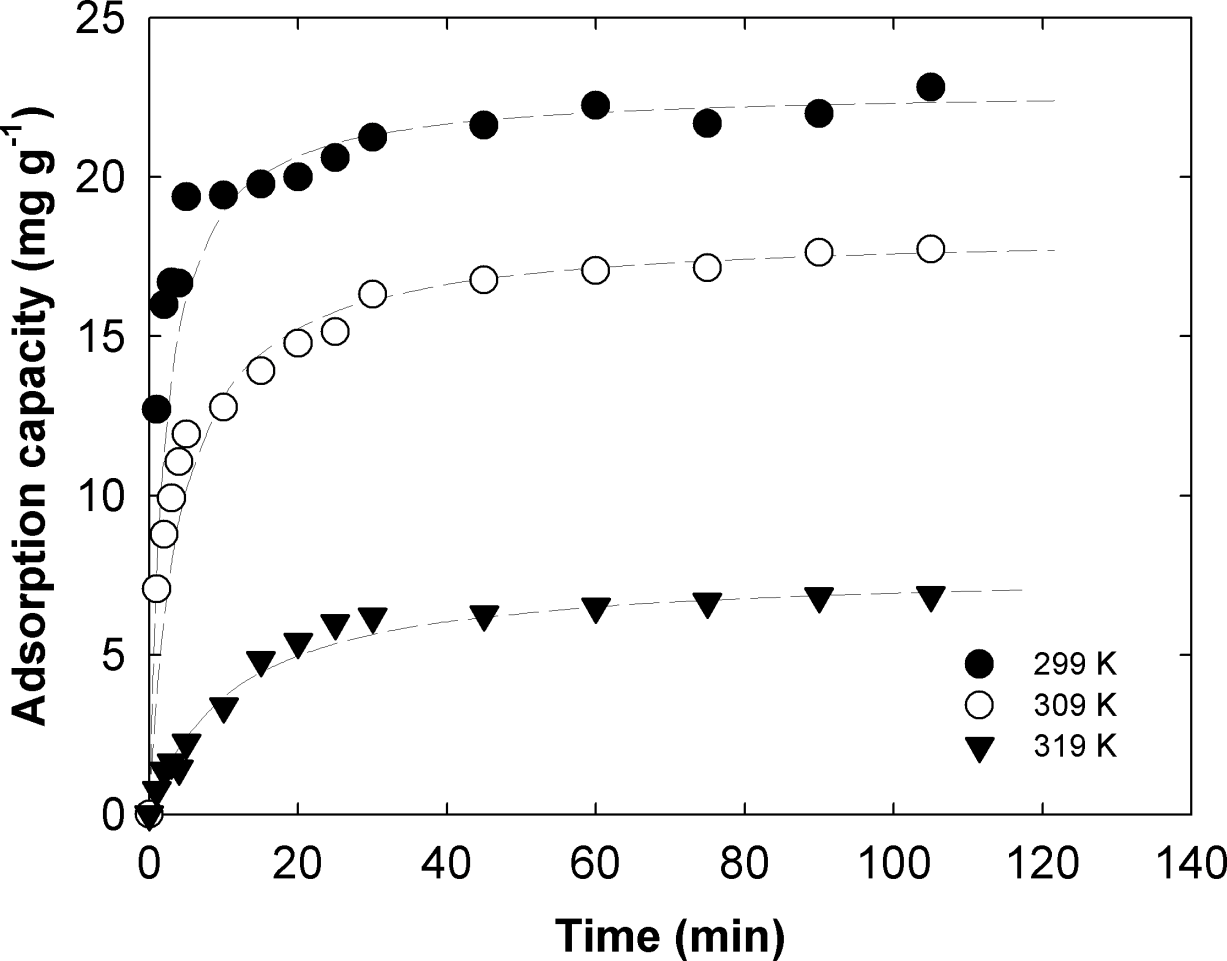
Temperature effect of IBP adsorption onto MSM-500.

Fig 3 shows kinetic data of IBP adsorption at 299, 309, and 319 K, and fit lines of the pseudo-second-order kinetic model. The *q*_*eq*_ of IBP uptake by MSM–500 was 22.8 mg g^–1^ at 299 K, but decreased to 18.28 and 7.7 mg g^–1^ at 309 and 319 K, respectively. This phenomenon has been seen in several studies on removing various pharmaceuticals with silica-based media and multi-wall carbon nanotubes (MWCNTs) [15, 38–40]. Based on the calculations of thermodynamic parameters, the *∆*_*ads*_ *G* ^*0*^ values were –10.4, 1.01, and 11.3 kJ mol^–1^ at 299, 309, and 319 K, respectively. Thus, the IBP adsorption is a spontaneous physical process at room temperature, but becomes non-spontaneous at higher temperatures. Moreover, *∆*_*ads*_ *G* ^*0*^, *∆*_*ads*_ *H* ^*0*^, and *∆*_*ads*_ *S* ^*0*^ were also calculated for comparison with other references (Table 4).

**Table 4:** Thermodynamics parameters (*∆*_*ads*_ *H* ^*0*^ and *∆*_*ads*_ *S* ^*0*^) of IBP uptake by MSM-500 and comparison with other references.

Sample	Temp. (K)	EDC	*Δ*_*ads*_ *H* ^*0*^ *(kJ mol* ^*–1*^ *)*	*Δ*_*ads*_ *S* ^*0*^ *(J mol* ^*–1*^ *K* ^*–1*^ *)*	Reference
UMS^a^	290–313	Molsidomine	–15	–27	[15]
MMS^b^	290–313	Molsidomine	–44	–127	[15]
PhMS^c^	290–313	Molsidomine	–65	–186	[15]
β-CD^d^	298–323	IBP	–52.08	–98.22	[38]
MWCNT^e^	288–318	Bisphenol AF	–17.16	–53.7	[39]
MWCNT	278–333	Estrone	–14.16	–560	[40]
MWCNT	278–333	17β-estradiol	–12.08	–460	[40]
MWCNT	278–333	17a-ethinylestradiol	–10.11	–420	[40]
MSM500	299–319	IBP	–23.0	–0.07	This study

UMS^a^: unmodified silica

MMS^b^: mercaptopropyl grafted silica material

PhMS^c^: phenol-modified silica

β-CD^d^: beta cyclodextrin

MWCNT^e^: multi-wall carbon nanotube.

The calculated *∆*_*ads*_ *H* ^*0*^ was –23.0 kJ mol^–1^, indicating that the IBP adsorption onto MSM–500 is an exothermic reaction, and *∆*_*ads*_ *S* ^*0*^ was found to be –0.07 J mol^–1^ K^–1^. Accordingly, as a consequence of IBP adsorption onto the pores of MSM–500, the randomness of IBP decreased because the degree of freedom or mobility of IBP is limited compared with IBP in solution. The *∆*_*ads*_ *S* ^*0*^ value was much smaller than other references, indicating that less energy is needed to restore the system back to its original state. The negative *∆*_*ads*_ *H* ^*0*^ and *∆*_*ads*_ *S* ^*0*^ suggest that the removal process of IBP can be considered an enthalpy-driven process.

### Regeneration

**Fig 4:**
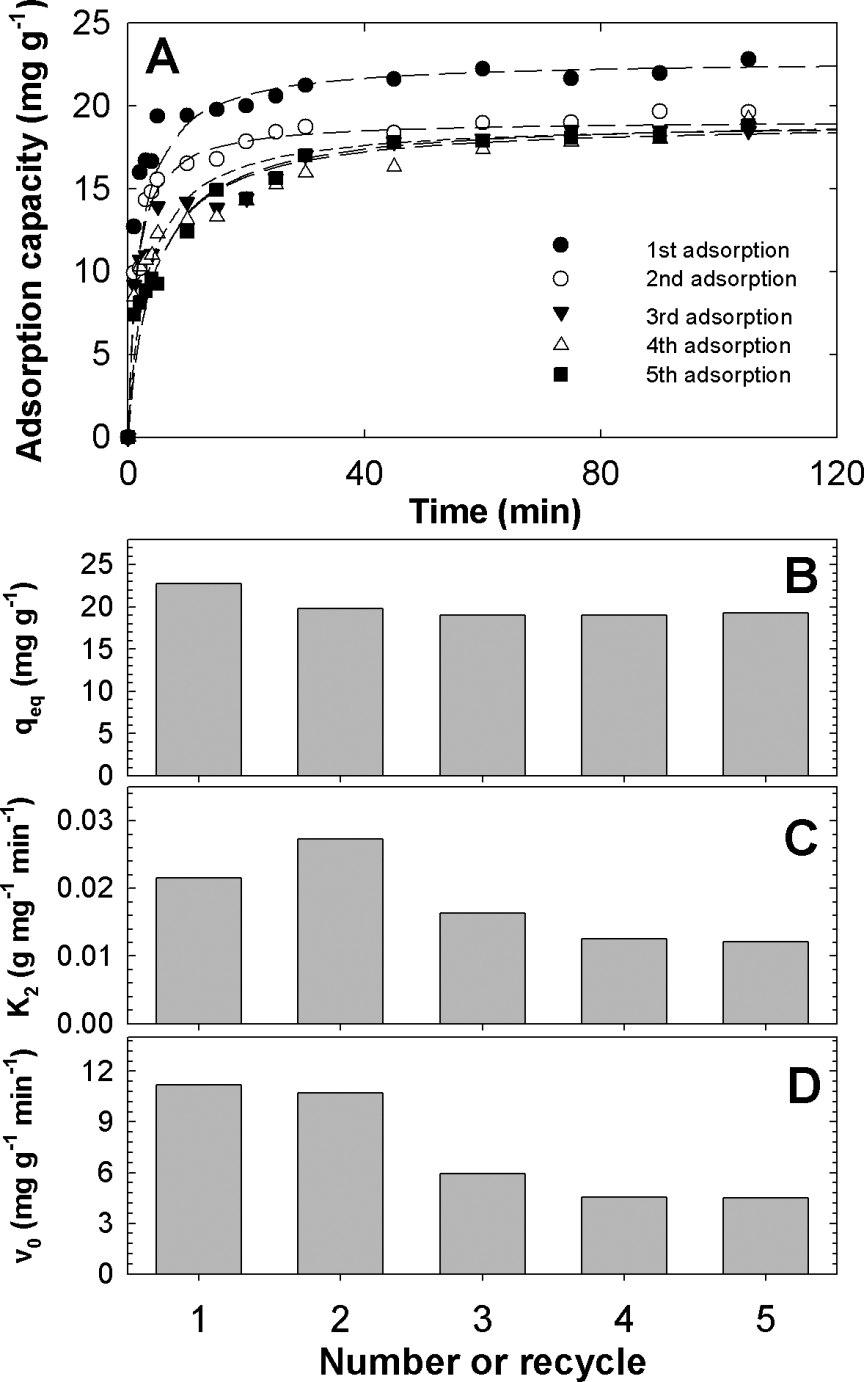
IBP uptake by MSM-500 at several recycles: (A) kinetics, (B) *q*_*eq*_, (C) *K*_*2*_, and (D) *v*_*0*_ according to the number of recycles.

Fig 4(A) presents kinetic data for five cycles of the adsorption of IBP by MSM–500 and fit lines of the pseudo-second-order kinetic model. Fig 4(B–D) show the *q*_*eq*_, *K*_*2*_, and *v*_*0*_, respectively, at each cycle. The first cycle had the highest *q*_*eq*_ (22.8 mg g^–1^). This result is similar to the kinetic tests conducted previously, suggesting that the experiments were reproducible under the same conditions. All consecutive cycles showed a *q*_*eq*_ of 19–19.8 mg g^–1^, slightly lower than the *q*_*eq*_ for the first cycle. Thus, based on the first *q*_*eq*_, MSM–500 had recovery efficiencies of 83–87%.

In the first cycle, 90% of the *q*_*eq*_ for IBP uptake was achieved at 5 min, but it slowed to ~30 min in the third and fourth cycles. In detail, the values of *K*_*2*_ and *v*_*0*_ were comparable in the first and second cycles, but dropped considerably at the third and decreased further at the fourth and fifth cycles. Accordingly, the reduction trends of IBP uptake capacity and speed were different, and these differences might have been affected by the deformation of the pore structure or the existence of some IBP molecules trapped in the pores after extraction with methanol. AC is composed mostly of micropores, and IBP molecules are adsorbed at the supermicropores, because its critical dimension is 0.72 nm [41]. Thus, extraction-based regeneration is not practical for spent AC due to the relatively high adsorption energy of AC. Generally, loaded AC is regenerated *ex situ* using heat or steam, which is a high energy-consuming process. Also, during this process, a large proportion of the AC can be lost [36]. In terms of regeneration, however, MSM–500 has advantages over AC due to simple and fast regeneration using methanol.

**Fig 5:**
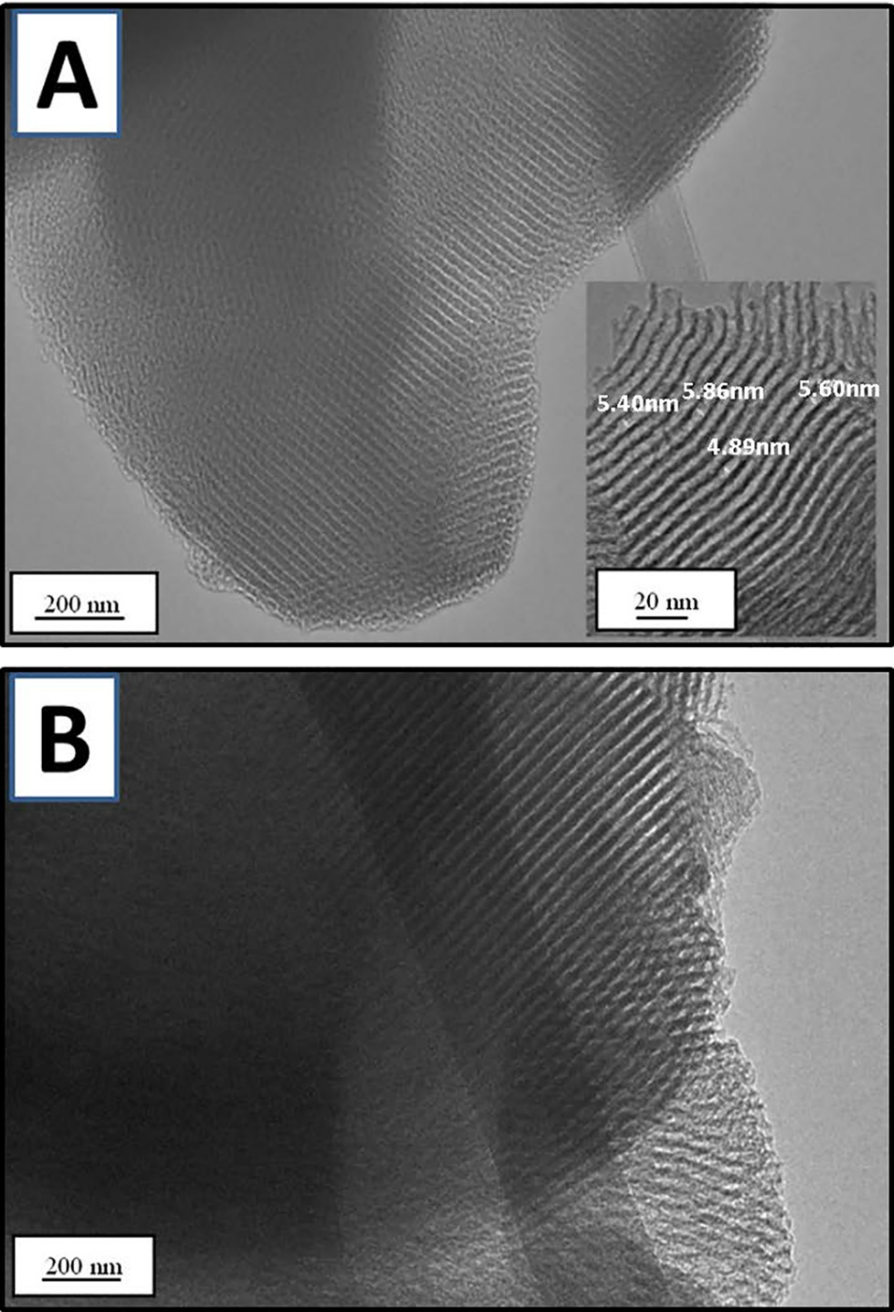
TEM images of MSM-500 (A) before and (B) after the fifth adsorption of IBP.

Fig 5 shows TEM images of MSM–500 before and after the fifth adsorption of IBP. As shown in Fig 5(A), MSM–500 had homogeneous pore and wall structures. The measured pore size was in the range of 5.44 ± 0.4 nm, ~11% smaller than the primary pore size (6.2 nm) as determined by the N_2_ gas isotherm. The TEM image of MSM–500 obtained after the fifth adsorption showed slight deformation at the inlet side of the pore structure, but no major defect. Thus, this result may be linked to the kinetic results for re-adsorption.

### Mechanism of adsorption of IBP by the MSMs

**Fig 6:**
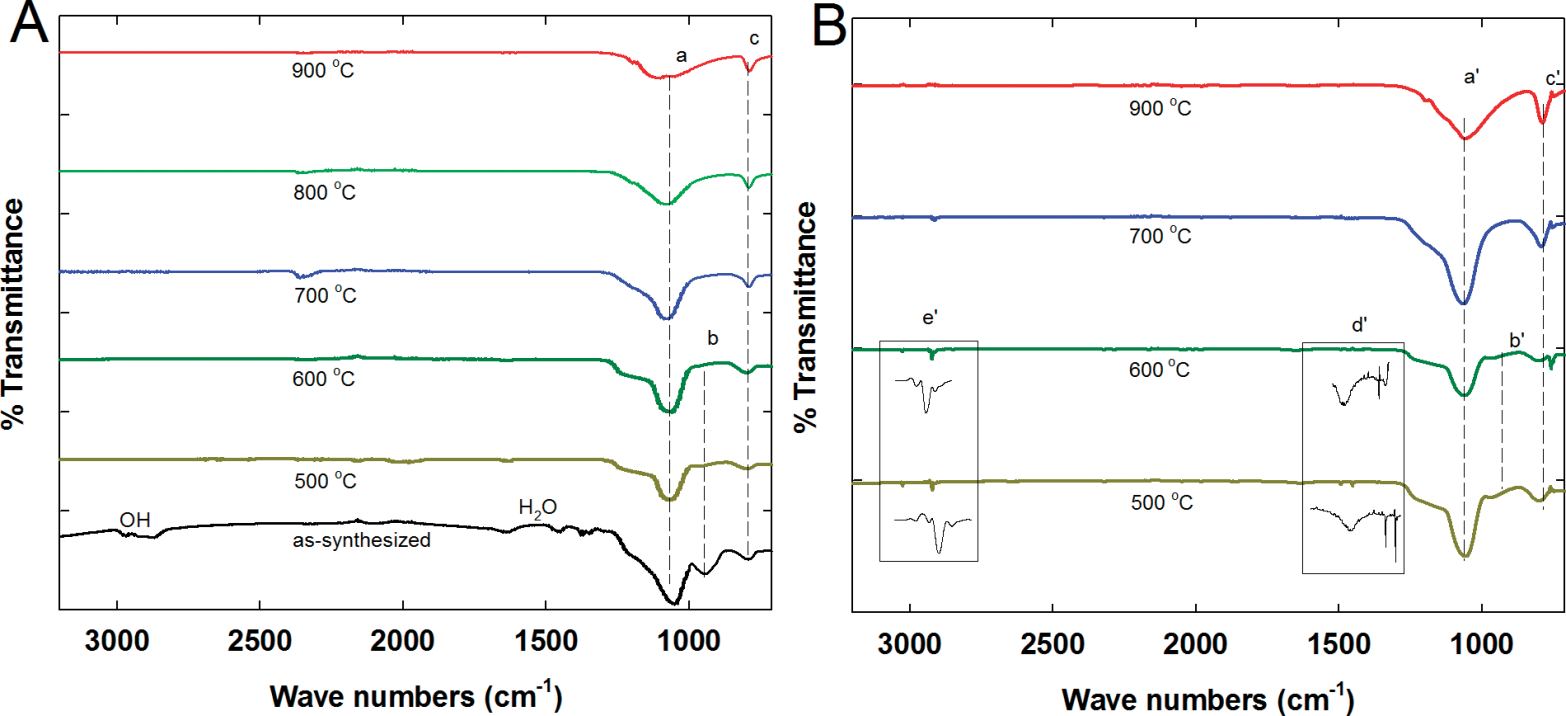
FTIR spectra of MSM materials before (A) and after adsorption (B) of IBP.

Fig 6 shows the FTIR spectra (4,000–500 cm^–1^) of all the MSMs prepared at different calcination temperatures, as well as those of IBP-retained MSMs. The as-synthesized MSM had obvious bands for OH (~3,400 cm^–1^), H_2_O (1,644 cm^–1^), Si–O–Si (symmetric stretching vibrations (*vs*.) at 1,057 cm^–1^ and ~800 cm^–1^), Si–OH (*vs*. at ~950 cm^–1^), and O–Si–O (deformation vibration (*δ*) at 435cm^–1^). Si–O–Si (lines a and c in Fig 6) and Si–OH (line b) of MSM-500 and -600 show FTIR spectra typical of MSMs [9]. The intensity of the Si–O–Si peak for the MSM calcined at >600°C decreased markedly, indicating dissociation of the Si–O–Si bond during calcination.

After the adsorption of IBP, the Si–O–Si (lines a′ and c′ in Fig 6) and Si–OH (line b′) peaks were preserved in the MSMs, showing stability of the pore structure. A new band, centered at 1,630 cm^–1^ (box labeled as d′), can be attributed to the asymmetric vibration of the carboxylic functional group (COO^–^). Two more sharp peaks, at 1,435 and 1,500 cm^–1^, were assigned to the C–H bonding of carbon atoms. The peak at 2,920 cm^–1^ (box labeled as e′) was assigned to a strong C–H bond, confirming the adsorption of IBP into the channels of MSM–500 [8, 9, 42]. In contrast, MSM–600 had a weaker adsorption peak, as shown in the d′ and e′ boxes, while the other MSMs showed no peaks in this range.

**Fig 7:**
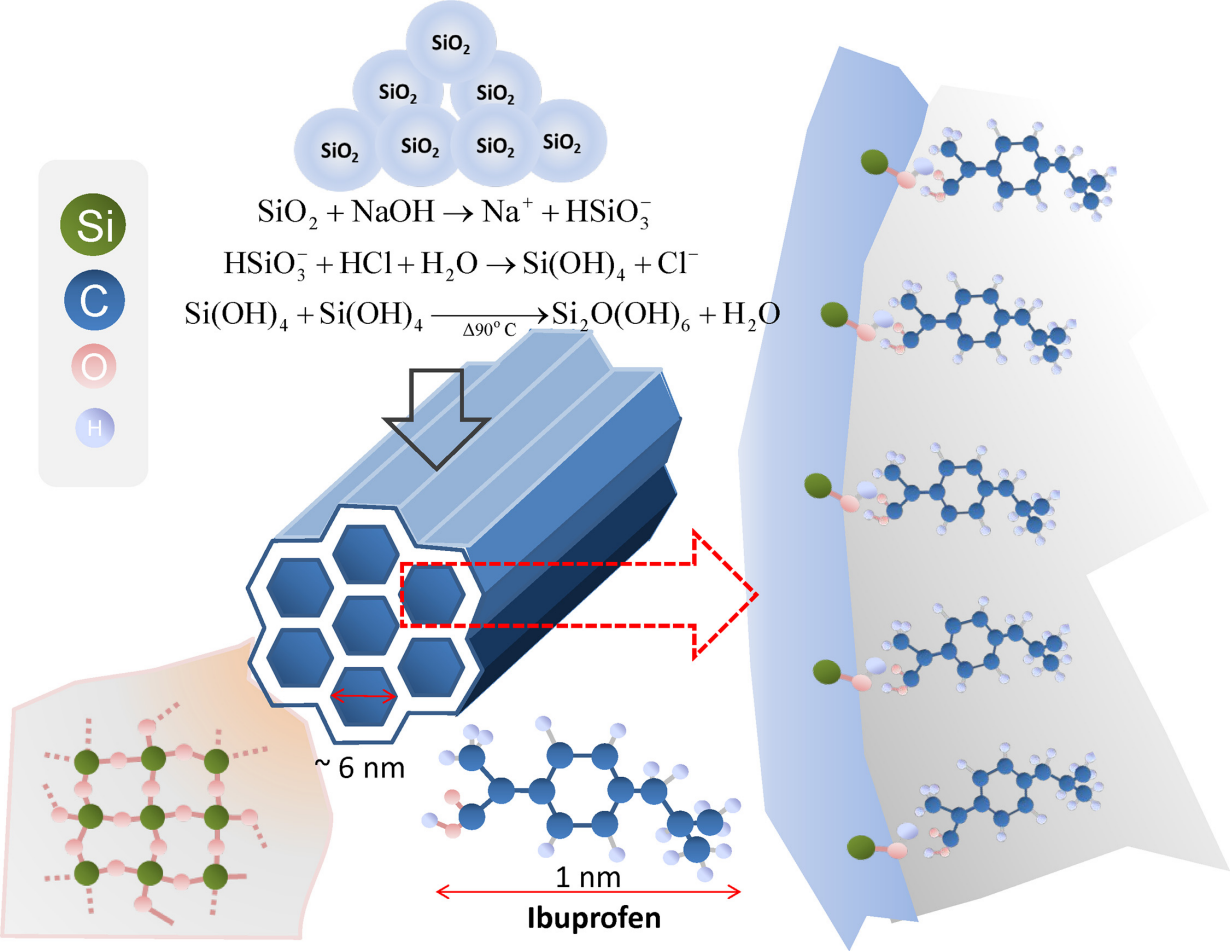
Mechanism of MSM synthesis and IBP adsorption.

These FTIR data suggest that the reaction mechanism between IBP and MSM is a hydrophilic reaction [1]. At pH 7, the hydrogen of the carboxylic group of IBP is dissociated to form a COO^−^functional group. The formation of a hydrogen bonding reaction between the COO^−^of IBP and silanol groups on the pore surface is favorable because it requires a lower activation energy [1, 7],

$$\equiv \text{Si}-\text{OH}+\;{}^{-}\text{OOC}-\text{B}\cdots \text{CH}\to \equiv \text{Si}-\text{O}\cdots \text{H}\cdots \text{OOC}-\text{B}\cdots \text{CH}$$

where ≡Si–OH and−OOC–B···CH are silanol and IBP, respectively. Fig 7 shows the overall mechanisms of MSM synthesis and IBP adsorption.

### IBP loading and release by the MSMs

**Fig 8:**
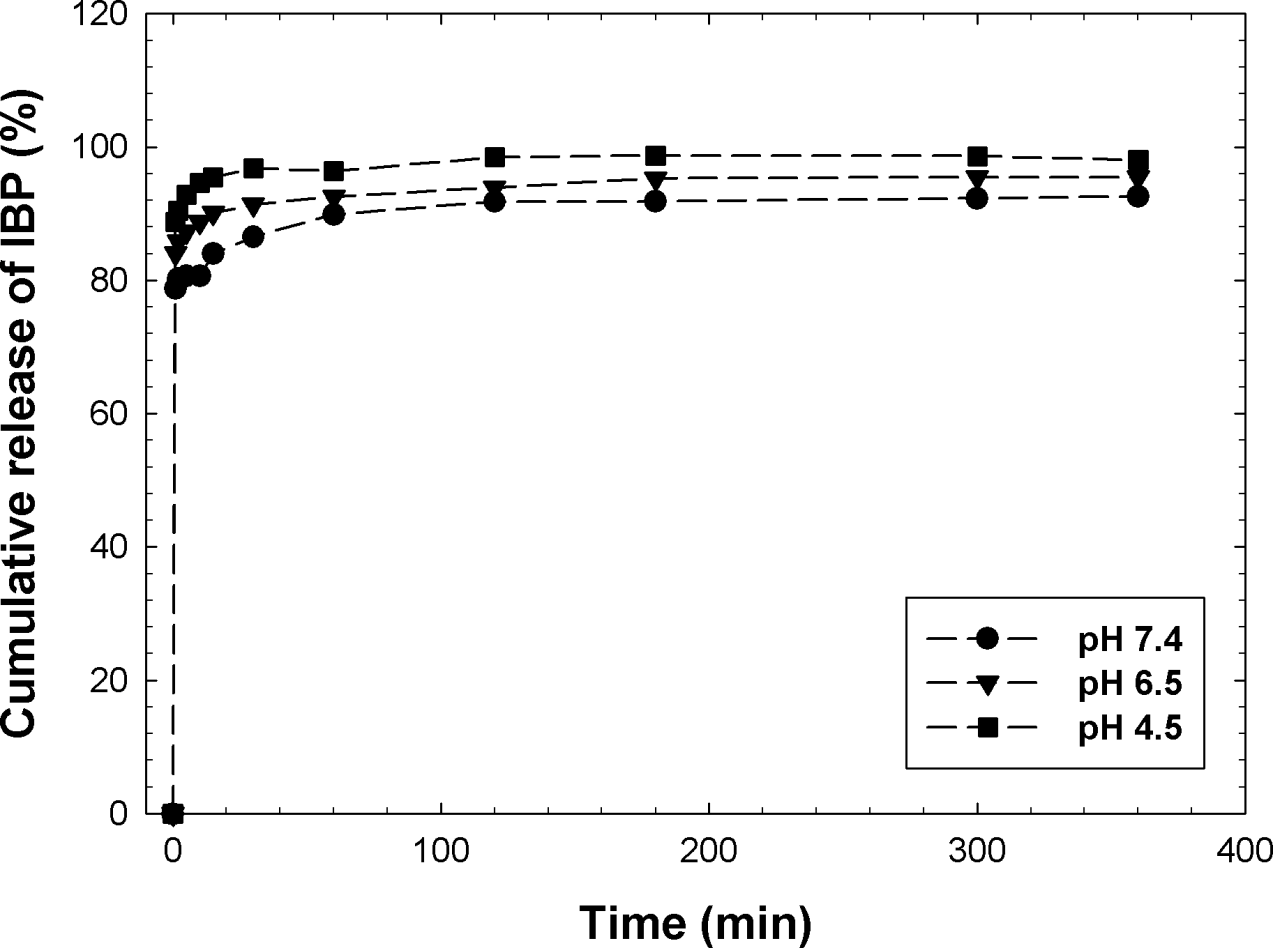
*In vitro* release of IBP from MSM-500 at different pH.

MSM–500 showed a 41% IBP drug loading capacity, comparable to the value reported for SBA–15 (20–30%) [6]. Fig 8 shows the *in vitro* drug release of the MSMs: almost 100% released within a few hours. Compared with other mesostructured materials, such as SBA–15 (~10 h), the unloading time of MSM–500 was much shorter. MSM–500 has a larger pore size than SBA–15 and MCM–41, which increases the diffusion of IBP molecules through the pores and subsequently increases the loading and unloading rates. The release behavior was also affected by the pH of the solution. At a lower pH, the IBP release was faster and higher, which is advantageous because most tumor cells in the body are slightly acidic and the drug release can be more specific to such target areas. Moreover pure SiO_2_ was utilized as a precursor, instead of TEOS to synthesis MSMs. This is expected to produce less toxic materials than the conventional mesoporous materials. Nakashima et al. have reported the acute and subchronic inhalation toxicity of TEOS in the synthesis of SBA–15, MCM–41 and MCM–48 [43].

### IBP removal and drug loading cost analysis

In this study, the initial synthesis costs of materials were calculated to estimate the removal or delivery costs for IBP, for which the IBP concentration and volume were 10 mg L^–1^ and 1 m^3^, respectively. As shown in Table 5, the MSM had the lowest cost ($0.76) *vs*. other silica-based mesoporous media.

**Table 5:** Comparison of pore characteristics and cost analyses for IBP removal.

Materials	Specific surface	Pore vol.	Conc. IBP	t	Q_max_	pH	^1^Mass	^2^Costs	Reference
	area	(cm^3^ g^–1^)	(mg L^–1^)	(min)	(mg g^–1^)		(g)	(USD)	
	(m^2^ g^–1^)								
Granular SBA– 15	767	0.86	0.1	1400	15	7	667	51.2	[44]
^3^MSN_450_	817	1.74	200	420	98.3	7	102	8.22	[45]
MSM	310	0.541	150	5–10	64.8	7	154	0.76	This study

^1^Mass of media required for treating 1 m^3^ of water containing IBP (10 mg L^–1^)

^2^Costs (USD) of media for treating 1 L of IBP containing water (10 mg L^–1^), which does not include electricity consumption, personnel, or regeneration.

^3^MSN_450_: mesoporous silica nanoparticles synthesized with 450 W of microwave power.

For example, the MSM was 67 times cheaper than granular SBA-15. The reduction in cost was mainly due to the replacement of TEOS with the much cheaper silicon source, SiO_2_. The allocated price of TEOS is ~96% of the total costs for synthesis and TEOS is ~200 times more expensive than SiO_2_. Furthermore, the reusability of MSM will further reduce the cost significantly.

## Conclusions

Adsorption isotherms and kinetics revealed that the MSMs synthesized showed relatively higher adsorption capacities and faster adsorption rates than other comparable media. The adsorption of IBP by MSM–500 was thermodynamically favorable at room temperature, but it involved relatively weak bonding because the calculated entropy was much smaller than in other references. In consecutive sorption studies, MSM–500 showed 83–87% recovery efficiencies, although it had a slower pattern of uptake. Based on the FTIR results, the reaction mechanism was identified as a hydrophilic interaction between the COO^−^of IBP and Si–OH of the pore surface. MSM–500 was also found to be suitable for IBP drug loading and unloading purposes, with a 41% loading capacity and almost 100% unloading efficiency within a few hours. The MSM was synthesized successfully using SiO_2_, so that its production costs were much lower than that of SBA-15. Thus, the new synthesis route developed in this study could have high potential for cost-effective mass production, and the MSM could be a suitable medium for industrial applications (for water treatment and drug delivery), as shown in the cost analysis study [6, 46, 47]. Furthermore, the use of inert precursor SiO_2_ is expected to produce less toxic MSMs, which are more suitable for water treatment and biomedical application.
